# Compound heterozygous *REC114* variants in dizygotic twins causes meiotic arrest and non-obstructive azoospermia

**DOI:** 10.1186/s12610-025-00291-0

**Published:** 2025-10-30

**Authors:** Wanze Ni, Chenwang Zhang, Shuai Xu, Wenbo Li, Dewei Qian, Haowei Bai, Yifan Sun, Zizhou Meng, Na Li, Chencheng Yao, Zheng Li, Peng Li, Yuxiang Zhang

**Affiliations:** 1 Department of Andrology, The Affiliated Taizhou People’s Hospital of Nanjing Medical University, Taizhou School of Clinical Medicine, Nanjing Medical University, Taizhou, 225300 China; 2 State Key Laboratory of Reproductive Medicine and Offspring Health, Nanjing Medical University, Nanjing, 211166 China; 3 Department of Andrology, the Center for Men’s Health, Urologic Medical Center, Shanghai Key Laboratory of Reproductive Medicine, Shanghai General Hospital, Shanghai Jiao Tong University School of Medicine, Shanghai, 200080 China

**Keywords:** Non-obstructive azoospermia, Meiosis, *REC114* gene, Double-strand break, Male infertility, Azoospermie non obstructive, Méiose, Gène REC114, Cassure double brin, Infertilité masculine, Génétique humaine

## Abstract

**Background:**

Meiosis is essential for gametogenesis and the maintenance of fertility. Central to this process is meiotic recombination, a mechanism that ensures accurate chromosome segregation and drives genetic diversity. Non-obstructive azoospermia (NOA), a severe form of male infertility, often results from meiotic arrest. Although monogenic variants in genes critical for meiosis have been identified as a cause, the etiology of approximately 60–70% of NOA cases remains unresolved, highlighting a significant gap in our understanding of its genetic basis.

**Case presentation:**

We utilized whole-exome sequencing (WES) to identify novel compound heterozygous variants in *REC114* (c.523_524del: p.Lys175GlufsTer50 and c.640_641del: p.Leu214SerfsTer11) in a pair of Chinese dizygotic twin brothers with NOA. These variants are predicted to generate a truncated REC114 protein and disrupt its interaction with MEI4, which is essential for the formation of double-strand breaks (DSBs) during meiosis. Testicular histopathology and meiotic chromosome spread analyses indicated meiotic arrest at the zygotene stage, consistent with a DSB formation defect.

**Conclusion:**

Our findings expand the spectrum of genetic factors contributing to NOA and suggest that the maintenance of DSB homeostasis is critical to the pathophysiology of meiotic arrest. This research offers new insights into the genetic basis of male infertility and holds potential for the development of improved genetic diagnostic tools.

**Supplementary Information:**

The online version contains supplementary material available at 10.1186/s12610-025-00291-0.

## Résumé

**Contexte:**

La méiose est essentielle à la gamétogenèse et au maintien de la fertilité. Au cœur de ce processus se trouve la recombinaison méiotique, un mécanisme qui assure une ségrégation chromosomique précise et favorise la diversité génétique. L’azoospermie non obstructive (ANO), une forme grave d’infertilité masculine, résulte souvent d’un arrêt méiotique. Bien que des variants monogéniques de gènes critiques pour la méiose aient été identifiés comme une cause, l’étiologie d’environ 60 à 70 % des cas d’ANO n’est toujours pas résolue, ce qui met en évidence une lacune importante dans notre compréhension de sa base génétique.

**Présentation des cas:**

Nous avons utilisé le séquençage de l’exome entier (WES) pour identifier de nouveaux variants hétérozygotes composés dans REC114 (c.523_524del : p.Lys175GlufsTer50 et c.640_641del : p.Leu214SerfsTer11) chez une paire de frères jumeaux dizygotes chinois avec ANO. On prédit que ces variants généreront une protéine REC114 tronquée et perturberont son interaction avec MEI4, qui est essentielle à la formation de cassures double brin au cours de la méiose. L’histopathologie testiculaire et les analyses de la propagation des chromosomes méiotiques ont indiqué un arrêt méiotique au stade zygotène, compatible avec un défaut de formation de cassures double brin.

**Conclusion:**

Nos résultats élargissent le spectre des facteurs génétiques contribuant à l’ANO, et suggèrent que le maintien de l’homéostasie de cassure double brin est essentiel à la physiopathologie de l’arrêt méiotique. Cette recherche offre de nouvelles perspectives sur les bases génétiques de l’infertilité masculine, et offre un potentiel pour le développement d’outils de diagnostic génétique améliorés.

## Introduction

Non-obstructive azoospermia (NOA) is the most severe form of male infertility and is characterized by impaired spermatogenesis that prevents the production of mature spermatozoa [1]. NOA can be classified as Sertoli-cell-only syndrome (SCOS), maturation arrest (MA), or hypospermatogenesis. MA is defined by the cessation of spermatogenesis at a specific stage, most commonly during meiosis at the spermatocyte stage. Meiosis is a meticulously orchestrated process involving a single round of DNA replication followed by two successive rounds of cell division. The common causes of MA include Y chromosome microdeletions, chromosomal abnormalities, and monogenic defects [2]. Through whole-exome sequencing (WES), numerous monogenic variants associated with MA have been identified in genes such as *Spermatogenesis Associated 22* (*SPATA22*) [3], *Meiotic nuclear divisions 1* (*MND1*) [4], *Testis Expressed 11* (*TEX11*) [5], *MutS Homolog 4* (*MSH4*) [6], *MutS Homolog 5* (*MSH5*) [7], and *Shortage In Chiasmata 1* (*SHOC1*) [8]. Nevertheless, the genetic causes for the majority of human MA cases remain poorly understood, and expanding the mutational spectrum of known causative genes is critical for elucidating the etiology of this condition.

During meiosis, the formation of programmed DNA double-strand breaks (DSBs) is regulated by the SPO11-TOPOVIBL complex, which is located on the chromosome axis. This process is mediated by the pre-DSB recombinosome, an assembly of evolutionarily conserved, meiosis-specific proteins, including meiotic recombination protein (REC114), Meiotic double-strand break formation protein 1 (MEI1), Meiotic double-strand break formation protein 4 (MEI4), Ankyrin repeat domain 31 (ANKRD31), and Interactor of HORMAD1 (IHO1). These factors are thought to target the SPO11-TOPOVIBL complex to PRDM9-dependent DSB hotspots, thereby ensuring DSB formation.

Studies in mice have demonstrated that the knockout of any single gene within this DSB formation complex is sufficient to disrupt DSB generation, leading to meiotic arrest and infertility. Pathogenic variants in DSB-related genes, including *REC114*,* MEI1*,* ANKRD31* and *MEI4*, have been implicated as causes of NOA and MA in humans [9–13]. Previous studies have reported both a biallelic loss‑of‑function (LoF) variant in *REC114* (c.568 C >T) and a compound heterozygous mutation (c.123 C >A/c.659_706dup) associated with NOA [14, 15]; however, additional compound heterozygous LoF alleles in this gene remain to be characterized.

In this study, we performed WES on two brothers from a Chinese family who were affected by NOA. We identified novel compound heterozygous LoF variants in *REC114* (c.523_524del: p.Lys175GlufsTer50 and c.640_641del: p.Leu214SerfsTer11). Our findings broaden the spectrum of known genetic variants underlying NOA, provide deeper insights into its genetic etiology, and may identify potential new targets for clinical therapy.

## Patients and methods

### Participants

Two brothers with idiopathic NOA were enrolled at the Department of Andrology, Urologic Medical Center, Shanghai General Hospital, Shanghai Jiao Tong University School of Medicine. They had no identifiable causes for azoospermia, such as varicocele, orchitis, cryptorchidism, testicular cancer, or a history of radiation or chemotherapy. A detailed family history was taken, and clinical assessments included semen analysis (according to the WHO 6th edition guidelines) [16], hormonal evaluation, and genetic testing, which excluded Klinefelter syndrome and AZF microdeletions. Both patients consented to WES and testicular biopsies for histopathological examination. The study was approved by the Institutional Ethical Review Committee of Shanghai General Hospital (Permit Number 2020SQ199). This study was designed as an in-depth, case-based characterization to establish a clear genotype-phenotype correlation, a necessary first step for interpreting novel variants discovered in rare genetic conditions.

### Whole-exome sequencing

Genomic DNA was extracted from peripheral blood samples using the DNeasy Blood and Tissue Kit (Qiagen). DNA integrity was evaluated with the Agilent 2100 Bioanalyzer, and libraries were prepared according to standard protocols. Paired-end sequencing (2 × 150 bp) was performed on an Illumina NovaSeq 6000 platform. The raw sequencing files were demultiplexed and converted to FASTQ format using bcl2fastq (v2.20). Quality control, including adapter removal and low-quality read trimming, was performed with fastp (v0.23.2). The sequences were aligned to the human reference genome (hg19/GRCh37) using the SpeedSeq pipeline. This reference build was chosen as it remains a robust standard in clinical genetics, offering extensive annotation and ensuring crucial compatibility with our established bioinformatics pipeline and key variant-filtering databases, such as the widely-used gnomAD v2.1.1, which is aligned to hg19. Potential artifacts, such as PCR duplicates and sample contamination, were assessed using VerifyBamID (v1.1.3). Variant calling for single nucleotide variants (SNVs) and small insertions/deletions (indels) was conducted with GATK’s UnifiedGenotyper (v3.8). ANNOVAR (v2019Oct24) was used to annotate variants with information from databases, including ClinVar, dbNSFP, and gnomAD (v2.1.1). Variants with a minor allele frequency exceeding 1% in any public database were excluded. Candidate variants were prioritized based on predicted pathogenicity. Missense variants were evaluated using SIFT, PolyPhen-2, and MutationTaster, whereas LoF variants were defined as truncating variants (nonsense/frameshift) or canonical splice-site alterations. Candidate genes were cross-referenced with testis-enriched expression profiles from the Human Protein Atlas and known infertility-associated genes from the OMIM and Mouse Genome Informatics databases. The final analysis was restricted to biallelic pathogenic variants following autosomal recessive or X-linked inheritance patterns.

### Sanger sequencing

Sanger sequencing was used to validate the *REC114* variants identified by WES. Genomic DNA was extracted from the peripheral blood of the brothers and their parents. The primers used for PCR and sequencing were as follows: REC114-F1: 5’-GCCTGTTTCGAGTACAGTTCAG-3’; REC114-R1: 5’- AAATGTCACACAGGTGCGTTC-3’; REC114-F2: 5’- CTATGTAAAGGAAGCCTGTAAAGC-3’; and REC114-R2: 5’- TTAATTTCTCAAACCCGCCAGC-3’. The PCR products were bidirectionally sequenced on a 3730xl DNA Analyzer (Applied Biosystems).

### Hematein-eosin (H&E) staining

Testicular biopsies were obtained from the NOA-affected brothers and from a patient with obstructive azoospermia (OA) who served as a control. The tissues were fixed in 4% paraformaldehyde, embedded in paraffin, and sectioned at a thickness of 5 μm. The sections were dewaxed, rehydrated, and stained with hematoxylin and eosin following the manufacturer’s protocol (ab245880, Abcam). Images were captured using a phase-contrast microscope (Leica SP8).

### Immunofluorescence (IF) staining

Testicular tissue sections were dewaxed and rehydrated. Antigen retrieval was performed in sodium citrate buffer (10 mM, pH 6.0) under high-temperature pressure. The sections were blocked with 10% BSA and incubated overnight at 4 °C with the following primary antibodies. These antibodies were selected as they are well-established markers for key meiotic events and structures, and their specificity has been extensively validated in previous studies of human spermatogenesis [4, 14]. Specifically, we used: mouse anti-γH2AX (1:300, 05–636, Millipore) to label DNA double-strand breaks; rabbit anti-DMC1 (1:100, SC-53269, Santa Cruz) as a marker for strand invasion during recombination; goat anti-SYCP3 (1:25, AF3750, R&D Systems) to visualize the axial elements of the synaptonemal complex; and peanut agglutinin (PNA, 1:400, Thermo Fisher) to identify acrosomes in spermatids After washing, the sections were incubated with species-specific Alexa Fluor^®^ 488/594-conjugated secondary antibodies, including donkey anti-goat AF488 (1:1000, A32814, Invitrogen), goat anti-mouse AF488 (1:1000, A11001, Invitrogen), and goat anti-mouse AF594 (1:1000, A11005, Invitrogen). Nuclei were counterstained with Hoechst 33342 (1:1000, 62249, Thermo Fisher). Images were acquired via a Leica SP8 confocal microscope. Sections were analyzed in a blinded manner by two independent observers. All experiments were performed in triplicate to ensure reproducibility.

#### In Silico analyses

The evolutionary conservation of the REC114 protein sites affected by the variants was assessed using the UniProt database. The potential structural impact of the variants on the IHO1-REC114-MEI4 complex was modeled with AlphaFold3 and visualized using UCSF ChimeraX 1.4 [17]. The model was built with the canonical protein sequences for IHO1 (Q8IYA8), REC114 (Q7Z4M0), and MEI4 (A8MW99) from UniProt [18, 19]. Subsequently, hydrogen bond analysis was performed to predict conformational changes induced by the variants.

## Case presentation

### Clinical findings

The proband and his dizygotic twin brother were 32 years old at the time of analysis. The family history was negative for consanguinity and fertility problems, and the siblings had no history of chronic diseases. Physical examination revealed normal development of the penis, scrotum, vas deferens, and epididymis. Testicular volumes were 12 mL bilaterally for both individuals. Semen analysis confirmed a normal volume but complete azoospermia. Both brothers had a 46,XY karyotype with no Y chromosome microdeletions. They had no history of cryptorchidism, hypogonadism, cancer, drinking, or smoking. Both underwent microdissection testicular sperm extraction (mTESE), but no sperm were found. Histopathological analysis of testicular tissue yielded a Johnsen score of 5 for both brothers, indicating NOA with a meiotic defect. Detailed information is shown in Table 1.

**Table 1:** Clinical characteristics of NOA patients with variants in *REC114*

	Proband	Brother	Reference
Age (years)	32	32	/
Height (cm)	175	170	/
Weight (kg)	70	70	/
Testis volume (left, mL)	12	12	12–15
Testis volume (right, mL)	12	12	12–15
Karyotype	46,XY	46,XY	46,XY
Y chromosome microdeletions	Normal	Normal	Normal
Semen volume (mL)	2.8	4.0	≥ 1.5
FSH (IU/L)	5.63	9.60	1.27–19.26
LH (IU/L)	2.05	2.41	1.24–8.62
T (µg/L)	3.07	2.38	1.75–7.81

*NOA* non-obstructive azoospermia, *FSH* follicle-stimulating hormone, *LH* luteinizing hormone, *T* testosterone

### Identification of the compound heterozygous variants of REC114 in the NOA-affected siblings

**Fig. 1:**
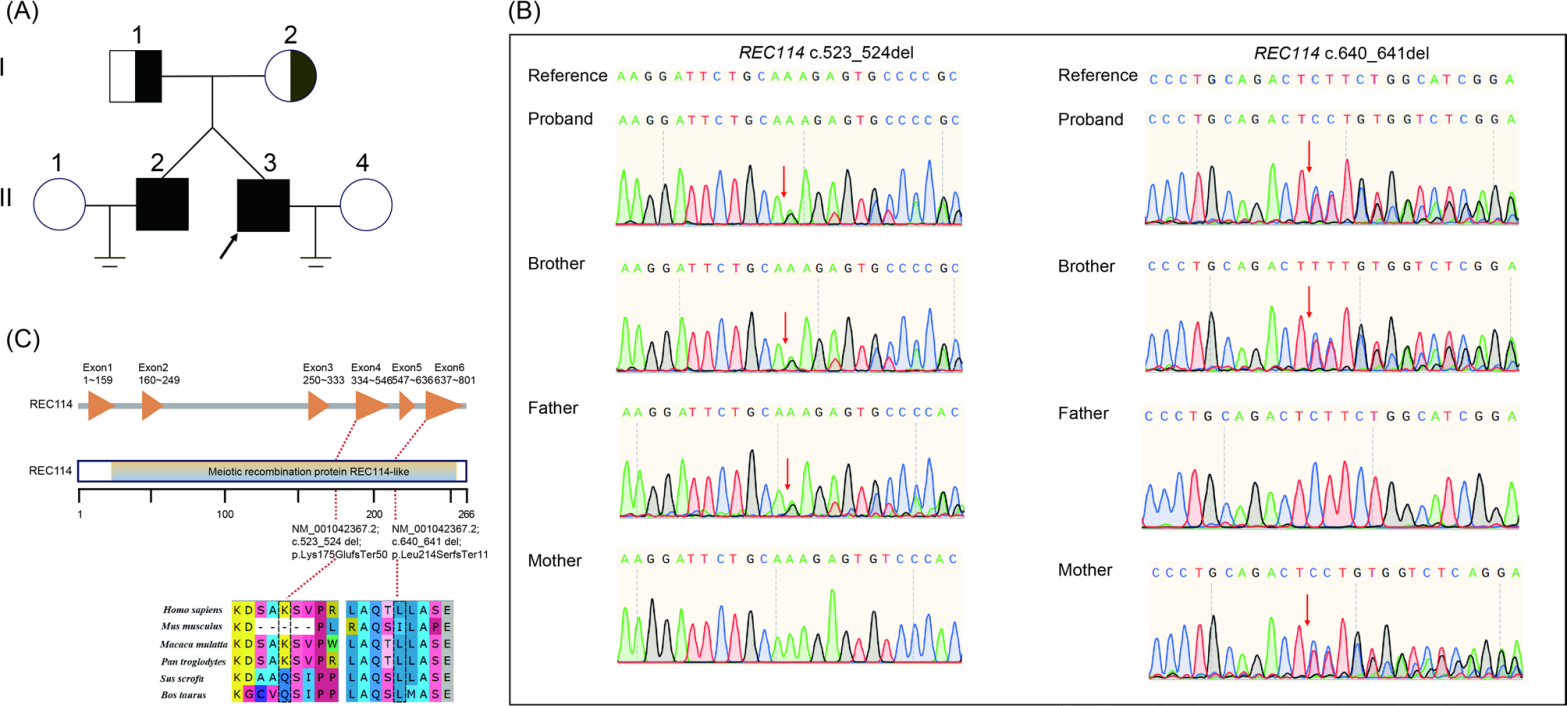
Characterization of Compound Heterozygous *REC114* Variants in NOA-affected Individuals. **A** Family pedigree and genotyping of the siblings with NOA. **B** Confirmation of *REC114* variants via Sanger sequencing. **C** The positions of frameshift variants in *REC114* are shown. The conservation of the *REC114* variant was analyzed

WES was performed on the proband and his brother. The pedigree is shown in Fig. 1A. Following data analysis, compound heterozygous variants in *REC114* (NM_001042367: c.523_524del: p.Lys175GlufsTer50 and c.640_641del: p.Leu214SerfsTer11) were identified as the most likely pathogenic variants associated with their meiotic defect. Sanger sequencing confirmed these compound heterozygous LoF variants in both brothers (Fig. 1B). The frequencies of these two variants in the gnomAD database are 0.000029020 and 0.000004924, respectively, and predictive tools such as MutationTaster and CADD classify them as deleterious (Table 2). Consistent with an autosomal recessive inheritance pattern, the c.523_524del variant was inherited from their father, and the c.640_641del variant was inherited from their mother. These frameshift variants are predicted to result in the absence of the MEI4-binding domain (amino acids 203–254), which is conserved across yeast, mice, and humans.

**Table 2:** Identification of compound heterozygous frameshift variants in the *REC114* gene among NOA patients

	Genetic Variant 1	Genetic Variant 2
*REC114* variant identified		
DNA change	c.523_524del	c.640_641del
Protein alteration	p.Lys175GlufsTer50	p.Leu214SerfsTer11
Variant allele	Heterozygosity	Heterozygosity
Variant type	Frameshift	Frameshift
Reference SNP ID	rs769571961	rs781734668
Allele frequency in human population		
All individuals in gnomAD V3.1.1	0.000029020	0.000004924
East Asians in gnomAD V3.1.1	NR	NR
1,000 Genomes Project	NR	NR

NCBI reference sequence number of *REC114* in GenBank: NM_001042367.2

*NOA *non-obstructive azoospermia, *SNP* single-nucleotide polymorphism, *NR* not reported

### Patients with the REC114 variant exhibit an MA phenotype

**Fig. 2:**
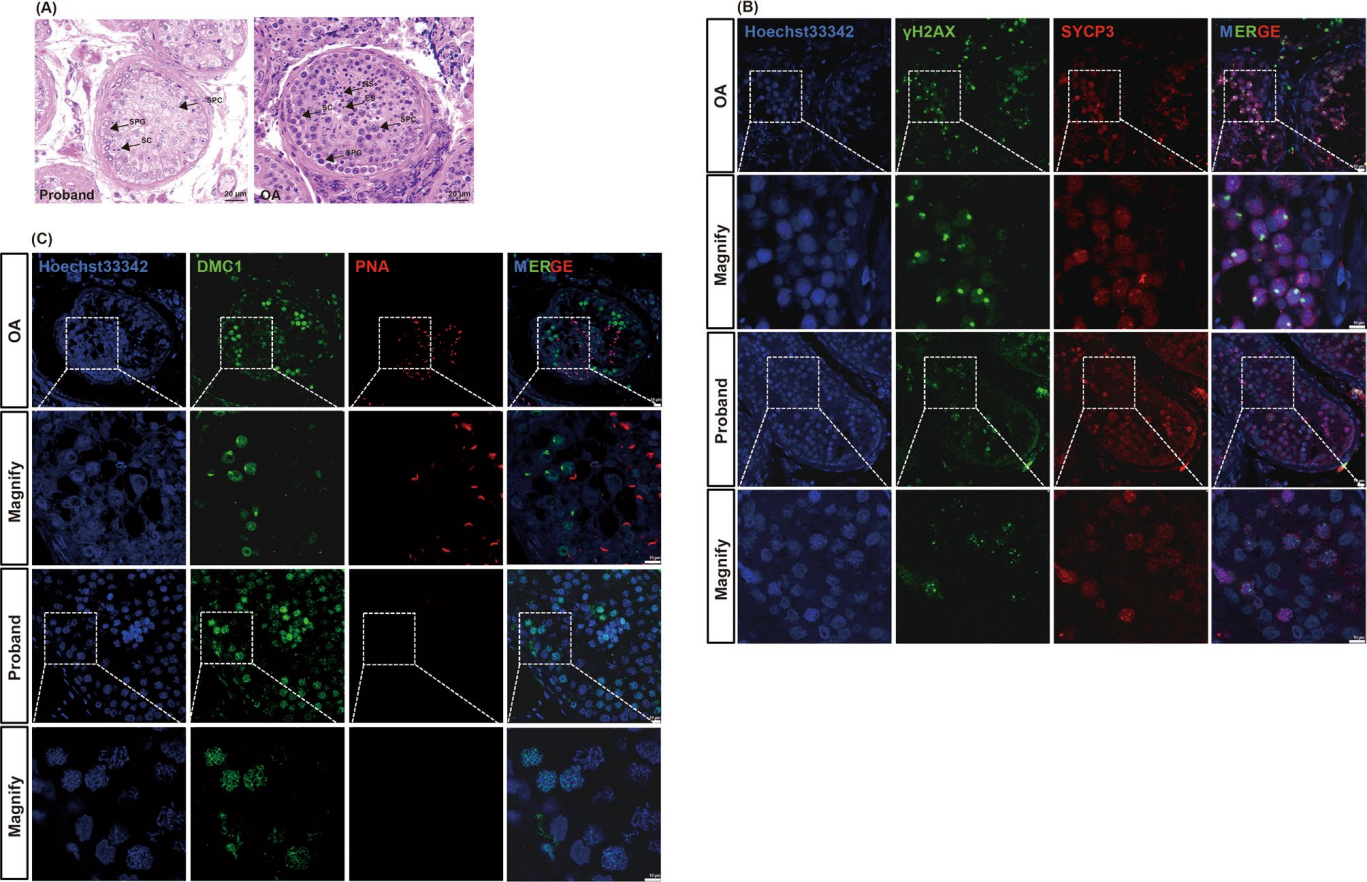
Spermatocyte arrest phenotype in the proband. **A** H&E staining of cross-sections of testicular biopsies from the proband and a patient with obstructive azoospermia (OA, positive control). SPG, spermatogonial; SPC, spermatocyte; RS, round spermatid; ES, elongated spermatid; SC, Sertoli cell.** B** IF staining showing the expression of γH2AX (green) and SYCP3 (red) in the testis of the proband and positive control. **C** IF staining showing the expression of DMC1 (green) and PNA (red) in the testis of the proband and positive control

The MA phenotype in the affected patients was investigated via H&E staining, IF, and meiotic chromosomal spread analysis. Compared with the control testis from a patient with OA, H&E staining of the proband’s testis revealed a reduced number of spermatocytes and a complete absence of spermatids and spermatozoa, whereas the numbers of Sertoli cells and spermatogonia were normal (Fig. 2A).

IF analysis revealed positive SYCP3 expression in the proband, but the XY body, typically labeled by γH2AX in pachytene, was not observed, indicating impaired DSB repair (Fig. 2B). Furthermore, the absence of a PNA signal confirmed that no spermatids or spermatozoa were present (Fig. 2C). In contrast, the expression of SYCP3, γH2AX, DMC1, and PNA appeared normal in the OA control tissue (Fig. 2B, C). These findings collectively indicate MA in patients.

**Fig. 3:**
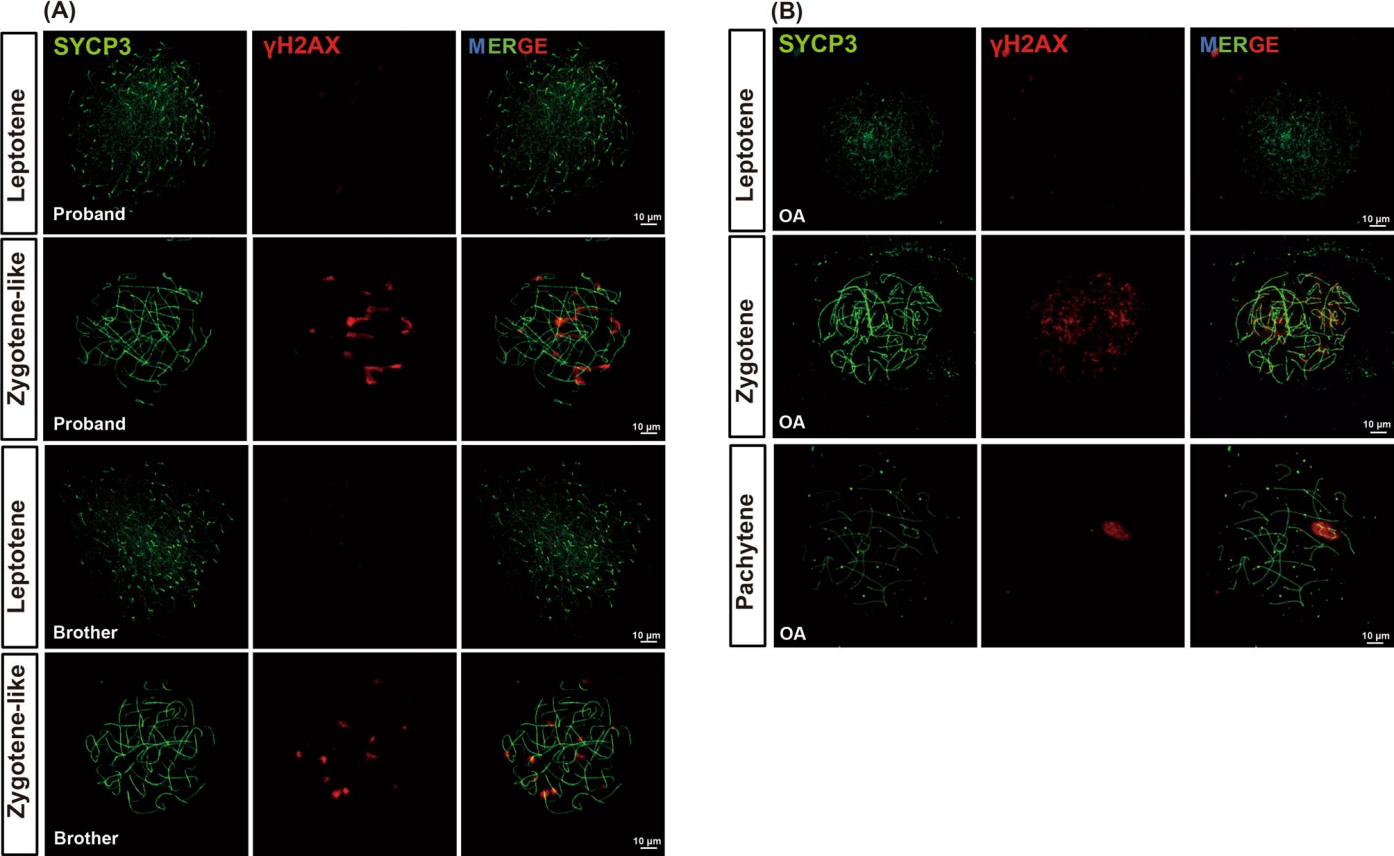
Meiotic spreads from the testis of the proband and the positive control. A meiotic chromosome spread assay revealed the expression of SYCP3 (green) and γH2AX (red) in the testes of the proband and his brother (**A**) and a patient with obstructive azoospermia (OA, positive control, **B**)

To pinpoint the specific stage of arrest, meiotic chromosomal spread analysis was performed. In the proband’s spermatocytes, γH2AX signals were observed at a relatively normal intensity but exhibited diffuse nuclear staining, and the X and Y chromosomes failed to condense to form the sex body. This pattern is indicative of MA at a zygotene-like stage (Fig. 3A). In contrast, complete progression of the meiotic stages from leptotene to pachytene was observed in the OA control (Fig. 3B). Therefore, the brothers with compound heterozygous LoF variants in *REC114* exhibit meiotic arrest at the zygotene-like stage.

### Analysis of REC114 expression and the structural changes caused by the identified REC114 variant

**Fig. 4:**
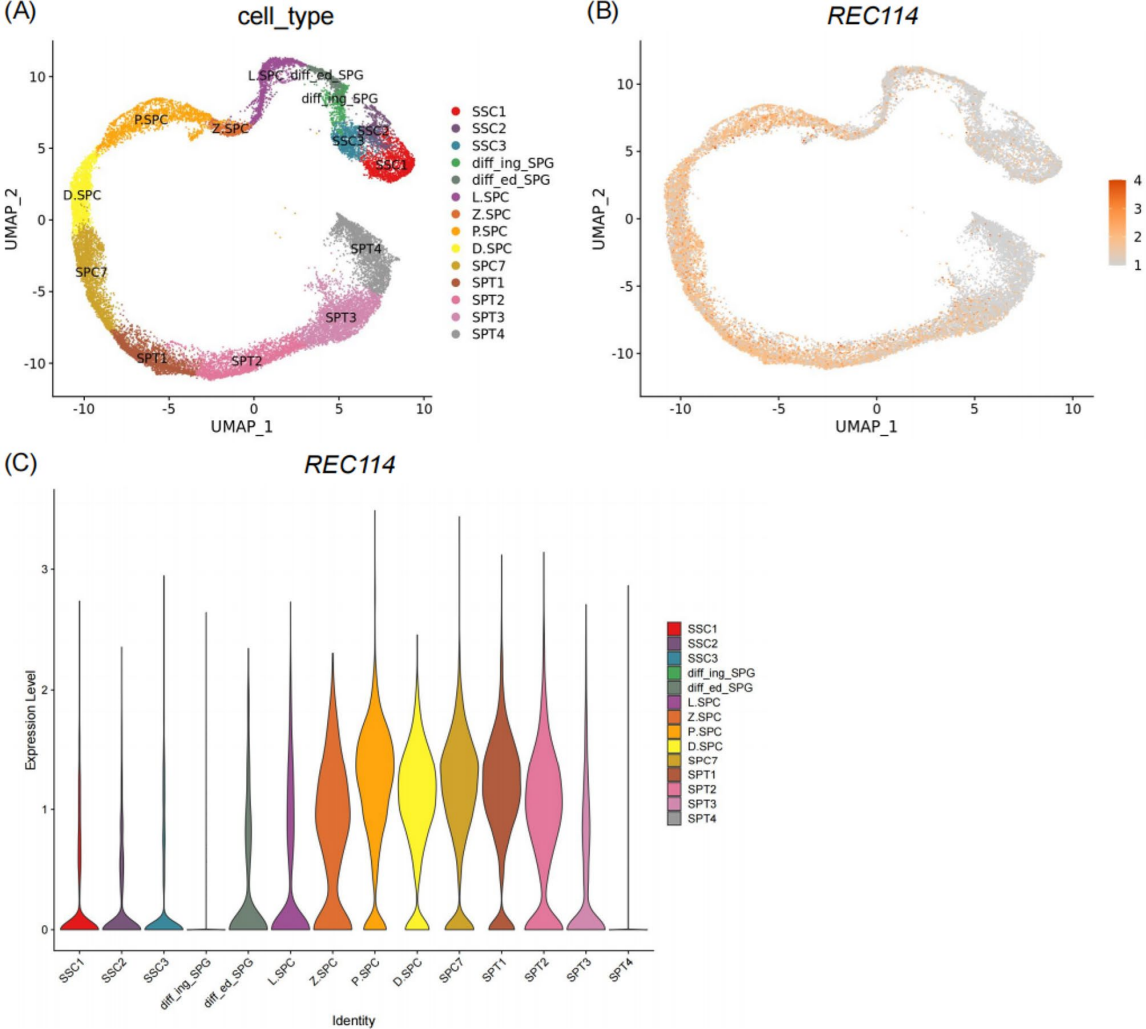
***REC114*** mRNA expression patterns in human testicular tissue. **A **UMAP plot of germ cell clusters detected by single-cell sequencing analyses in adult human testes. **B** UMAP expression pattern of *REC114* in human adult testicular germ cells. **C** Expression patterns (violin plot) of *REC114* across different human germ cell clusters. SSC1: spermatogonial stem cell I; SSC2: spermatogonial stem cell II; SSC3: an aligned spermatogonial III; diff_ing_SPG: differentiating spermatogonia; diff_ed_SPG: differentiated spermatogonia; L_SPC: leptotene spermatocyte; Z_SPC: zygotene spermatocyte; P_SPC: pachytene spermatocyte; D_SPC: diplotene spermatocyte; SPC7: a mixture of diakines, metaphase, anaphase, telophase, and secondary spermatocytes; progenitor, spermatogonial progenitor; SPT: spermatid

To investigate REC114 expression during human spermatogenesis, we analyzed single-cell RNA sequencing (scRNA-seq) data from the Male Health Atlas (MHA) [20]. The analysis revealed that *REC114* is highly expressed in meiotic prophase I spermatocytes, particularly at the zygotene stage, with transcripts persisting into the early spermatid stage (Fig. 4). The AlphaFold model of the IHO1-REC114-MEI4 complex predicted that the truncating variants in REC114 lead to structural alterations, accompanied by a significant reduction in contacts and hydrogen bonds with IHO1 and MEI4 (Supplementary Figure**S1**).

## Discussion

In this study, we identified compound heterozygous LoF variants in *REC114* (c.523_524del and c.640_641del) in dizygotic twin brothers with NOA. These variants are predicted to produce truncated REC114 proteins, a state consistent with the observed MA at the zygotene stage. This report provides additional evidence supporting the role of *REC114* as a candidate gene for MA and NOA.

*REC114*, located at chromosome 15q24.1, is a meiosis-specific gene that plays a crucial role in the formation of programmed DSBs during meiotic prophase I [14]. These DSBs are essential for homologous recombination and proper chromosome segregation. Meiotic DSBs are generated by the SPO11-TOPOVIBL complex, and accessory proteins such as REC114, MEI4, and IHO1 are key to regulating the activity of this complex [11, 12, 21]. REC114 interacts directly with MEI4, forming a pre-DSB recombinosome that helps target the SPO11-TOPOVIBL complex to DSB hotspots [12].

*Rec114*-knockout mice exhibit significant defects in DSB formation, resulting in early prophase meiotic arrest and infertility [14, 21]. The *REC114* variants identified in our study are predicted to produce a truncated protein lacking the C-terminal domain essential for interaction with MEI4, likely disrupting the REC114-MEI4 complex formation and thereby contributing to MA at the zygotene-like stage. Our IF staining results confirmed normal chromosome axis formation (SYCP3 expression) but diffuse nuclear γH2AX signals and a failure to form the sex body, which is consistent with this defect. Previous co-immunoprecipitation assays have shown that a similarly mutated REC114 protein fails to form a stable complex with MEI4, underscoring the importance of the REC114-MEI4-IHO1 axis in regulating DSB formation [14]. The conservation of these protein interactions across species highlights the evolutionary importance of REC114 in meiosis. Our findings align with results from mouse models and structural analyses, which together emphasize that the REC114-MEI4 interaction is critical for proper DSB formation and homologous recombination.

### Limitations of the study

This study has two main limitations. First, our findings are based on a single family, and validation in larger cohorts is needed. However, this in-depth case study aligns with ACMG guidelines for rare variant interpretation, providing crucial foundational evidence [22]. Second, our functional assessment relies on in-silico predictions and histopathology. While these results strongly suggest a pathogenic effect, definitive proof of causality requires further experimental validation. Future in vitro studies, such as protein interaction assays, and the development of in vivo models are necessary to fully elucidate the molecular mechanisms by which these variants disrupt meiosis.

## Conclusion

This study reports novel compound heterozygous variants in *REC114* associated with MA and NOA, further highlighting the crucial role of *REC114* in maintaining DSB homeostasis and ensuring proper meiotic progression. Our findings expand the mutational spectrum of *REC114* associated with male infertility and reinforce the genetic basis of this condition, which is crucial for improving the genetic diagnosis of NOA. Further research is needed to identify additional causative genes and elucidate their molecular mechanisms.

## Supplementary Information

Supplementary Material 1. Figure S1 Modeling of the human 4:4:2 IHO1-REC114-MEI4 complex and conformational changes caused by the *REC114* variant.The structure of the 4:4:2 IHO1‐REC114‐MEI4 complex predicted by AlphaFold3. The green chain represents IHO1, the purple chain represents MEI4, and the orange/red chain represents REC114. The black dashed box indicates hydrogen-bonding interactions.Magnified images of the black dashed boxes in Figureand Figure. The red dashed lines represent hydrogen bonds. The black arrow indicates the variant affected residue. The structure of the mutant REC114complex.Magnified image of the black, dashed box in Figure. The black dotted lines represent hydrogen bonds. The black arrows indicate missense amino acids. Red arrows indicate premature termination codons. The structure of the mutant REC114complex.Magnified image of the black, dashed box in Figure. The red dotted lines represent hydrogen bonds. The black arrows indicate missense amino acids. Red arrows indicate premature termination codons.

## Data Availability

The data that support the findings of this study are available upon request from the corresponding author. The data are not publicly available due to privacy or ethical restrictions.

## Abbreviations

DSBs: Double-strand breaks
H&E: Hematoxylin-eosin
IF: Immunofluorescence
LoF: Loss-of-function
MA: Maturation arrest
mTESE: Microdissection testicular sperm extraction
NOA: Non-obstructive azoospermia
OA: Obstructive azoospermia
SCOS: Sertoli-cell-only syndrome
scRNA-seq: Single-cell RNA sequencing
SNP: Single-nucleotide polymorphism
SNVs: Single nucleotide variants
WES: Whole-exome sequencing
